# Conditioned Variation in Heart Rate During Static Breath-Holds in the Bottlenose Dolphin (*Tursiops truncatus*)

**DOI:** 10.3389/fphys.2020.604018

**Published:** 2020-11-24

**Authors:** Andreas Fahlman, Bruno Cozzi, Mercy Manley, Sandra Jabas, Marek Malik, Ashley Blawas, Vincent M. Janik

**Affiliations:** ^1^Global Diving Research Inc., Ottawa, ON, Canada; ^2^Research Department, Fundación Oceanogràfic de la Comunidad Valenciana, Valencia, Spain; ^3^Department of Comparative Biomedicine and Food Science, University of Padua, Legnaro, Italy; ^4^Siegfried & Roy’s Secret Garden and Dolphin Habitat, The Mirage, Las Vegas, NV, United States; ^5^National Heart and Lung Institute, Imperial College London, London, United Kingdom; ^6^Department of Internal Cardiology Medicine, Faculty of Medicine, Masaryk University, Brno, Czechia; ^7^Nicholas School of the Environment, Duke University Marine Laboratory, Beaufort, NC, United States; ^8^Sea Mammal Research Unit, Scottish Oceans Institute, School of Biology, University of St Andrews, St Andrews, United Kingdom

**Keywords:** dive response, diving physiology, marine mammal, reflex, cardiovascular physiology, selective gas exchange hypothesis, adaptation, cardiovascular function

## Abstract

Previous reports suggested the existence of direct somatic motor control over heart rate (*f*_H_) responses during diving in some marine mammals, as the result of a cognitive and/or learning process rather than being a reflexive response. This would be beneficial for O_2_ storage management, but would also allow ventilation-perfusion matching for selective gas exchange, where O_2_ and CO_2_ can be exchanged with minimal exchange of N_2_. Such a mechanism explains how air breathing marine vertebrates avoid diving related gas bubble formation during repeated dives, and how stress could interrupt this mechanism and cause excessive N_2_ exchange. To investigate the conditioned response, we measured the *f*_H_-response before and during static breath-holds in three bottlenose dolphins (*Tursiops truncatus*) when shown a visual symbol to perform either a long (LONG) or short (SHORT) breath-hold, or during a spontaneous breath-hold without a symbol (NS). The average *f*_H_ (i*f*_Hstart_), and the rate of change in *f*_H_ (di*f*_H_/dt) during the first 20 s of the breath-hold differed between breath-hold types. In addition, the minimum instantaneous *f*_H_ (i*f*_Hmin_), and the average instantaneous *f*_H_ during the last 10 s (i*f*_Hend_) also differed between breath-hold types. The di*f*_H_/dt was greater, and the i*f*_Hstart_, i*f*_Hmin_, and i*f*_Hend_ were lower during a LONG as compared with either a SHORT, or an NS breath-hold (*P* < 0.05). Even though the NS breath-hold dives were longer in duration as compared with SHORT breath-hold dives, the di*f*_H_/dt was greater and the i*f*_Hstart_, i*f*_Hmin_, and i*f*_Hend_ were lower during the latter (*P* < 0.05). In addition, when the dolphin determined the breath-hold duration (NS), the *f*_H_ was more variable within and between individuals and trials, suggesting a conditioned capacity to adjust the *f*_H_-response. These results suggest that dolphins have the capacity to selectively alter the *f*_H_-response during diving and provide evidence for significant cardiovascular plasticity in dolphins.

## Introduction

In 1870, Paul Bert published his work showing a remarkable bradycardia associated with apnea in ducks from 100 beats ⋅ min^–1^ while breathing at the surface to 14 beats ⋅ min^–1^ while submerged (Bert, 1870). Following a number of studies, Irving summarized the cardiorespiratory adaptations in mammals that enable prolonged apnea (Irving, 1934, 1935; Irving et al., 1935; Irving, 1937, 1939; Irving et al., 1941a). Irving proposed that the cardiovascular changes, with a diving bradycardia and peripheral vasoconstriction, result in decreased cardiac output (CO) that would conserve the available O_2_ for hypoxia sensitive tissues such as brain and heart (Irving, 1939). In 1940, Scholander published his seminal studies that showed a significant diving bradycardia during forced dives (Scholander, 1940), and together with Irving continued to define the cardiovascular changes observed during diving in both animals and man (Scholander, 1940, 1963; Irving et al., 1941b).

Development of electronic devices that could record physiological changes continuously allowed the cardiovascular responses to be measured in freely diving birds and mammals. These studies confirmed that a diving bradycardia was observed during voluntary dives, but it was much more variable and not always as extreme as during forced dives (Elsner, 1965, 1966; Elsner et al., 1966; Kooyman and Campbell, 1972; Jones et al., 1973; Butler and Woakes, 1979; Kanwisher et al., 1981; Blix and Kjekshus, 1983; Blix, 1987; Ponganis et al., 1991; Thompson and Fedak, 1993; Andrews et al., 1997; Ponganis et al., 1997; Houser et al., 2010; Fahlman et al., 2019b). Based on these data, the significance of the dive response has been argued. Some have suggested that it may be an ancestral trait present in most taxa rather than a trait specific for diving (Blix, 1987; Hochachka and Mottishaw, 1998; Mottishaw et al., 1999), while others argued that the primary role of the diving bradycardia is to regulate the degree of hypoxia in skeletal muscle so that blood and muscle O_2_ stores can be used more efficiently (Davis and Kanatous, 1999). A different hypothesis was proposed in 2018, called the *selective gas exchange* hypothesis, proposing that breath-hold diving marine vertebrates have anatomical and physiological mechanisms that help regulate gas exchange (Hodanbosi et al., 2016; García-Párraga et al., 2018). The specific adaptations suggested for cetaceans included collateral ventilation, hypoxic pulmonary vasodilatation, and the ability to regulate cardiac output and blood flow distribution in proportion with the expected dive duration and depth (Olson et al., 2010; Hodanbosi et al., 2016; García-Párraga et al., 2018). The latter was based on past experiments where animals were conditioned to adjust *f*_H_ in response to an anticipated task following an auditory or visual stimulus. For example, in two harbor porpoises, it was shown that the *f*_H_-response was greater during 80 s breath-holds as compared with breath-holds for 20 s. However, this experimental design was not randomized, and animals first performed all of the longer dives before they completed the shorter breath-holds (Elmegaard et al., 2016). Similar studies on pinnipeds, comparing spontaneous as well as forced and voluntary dives also found differences that suggested a conditioned ability to vary *f*_H_ (Irving et al., 1941b; Elsner, 1965; Harrison et al., 1972; Ridgway et al., 1975), but these results could also be explained by different levels of stress or physical activity in different conditions. Without a comparison of the reaction to trained symbols for different dive durations, conditioned control cannot be ascertained.

To follow up on these previous studies with an experimental design that randomized the order of the dive duration, and to further evaluate the *selective gas exchange* hypothesis and the cardiorespiratory physiology in cetaceans, we wanted to determine the extent by which cetaceans are able to adjust *f*_H_, and thereby cardiac output (Bickett et al., 2019; Fahlman et al., 2019b, 2020b), based on the expected dive duration. For this purpose, we conditioned 3 bottlenose dolphins (*Tursiops truncatus*) to voluntary participate in static breath-holds. To determine whether dolphins could be conditioned to visually differentiate the expected breath-hold duration, and whether this would condition the *f*_H_-response, we separated the static breath-holds into SHORT (30 s) or LONG (120–180 s). For these two groups, the dolphin was shown a visual symbol which indicated the type of breath-hold they were to execute. We also measured the *f*_H_ response during voluntary static breath-holds in which the dolphin determined how long they would hold their breath. For this type of breath-hold, there was no visual symbol (NS) given before the breath-hold. If conditioning of the *f*_H_ was possible, we would expect a faster rate of change in *f*_H_ to a lower level when the dolphins anticipated a LONG breath-hold as compared to a SHORT one. We also hypothesized that voluntary breath-holds would be more variable and show the slowest rates of change in *f*_H_ with a much greater variation in the lowest *f*_H_.

## Materials and Methods

Three male bottlenose dolphins (*Tursiops truncatus*), housed in professional care at Siegfried and Roy’s Secret Garden Dolphin habitat in Las Vegas, Nevada, United States, participated in the study (Table 1).

**TABLE 1:** Animal ID, age (years in 2019), body mass (*M*_b_, kg), type of breath-hold [LONG, SHORT and No Symbol (NS)], number of trials (n), average (±s.d.) breath-hold duration [BHD (range), s], instantaneous heart rate before the breath-hold (pre-i*f*_H_), rate of change in instantaneous *f*_H_ (di*f*_H_/dt) during the first 20 s of the breath-hold, the average (i*f*_Hstart_) and minimum i*f*_H_ (i*f*_Hstartmin_) during the first 20 s of the breath-hold, the minimum instantaneous *f*_H_ (i*f*_Hmin_) during the breath-hold, or the average i*f*_H_ during the last 10 s of the breath-hold (i*f*_Hend_).

Animal	Age	*M*_b_ (kg)	Dive type	n	BHD (s)	pre-i*f*_H_ (beats ⋅ min^–1^)	log_10_(di*f*_H_/dt)	i*f*_Hstartmin_ (beats ⋅ min^–1^)	i*f*_Hstart_ (beats ⋅ min^–1^)	i*f*_Hmin_ (beats ⋅ min^–1^)	i*f*_Hend_ (beats ⋅ min^–1^)
D1	40	225	LONG	6	95 ± 47 (13–125)	85.0 ± 7.8	−0.47 ± 0.29	38.3 ± 4.0	62.0 ± 10.5	24.9 ± 0.8	31.8 ± 11.5
			SHORT	5	33 ± 2 (31–35)	87.8 ± 5.6	−0.35 ± 0.17	36.1 ± 0.7	63.5 ± 13.2	32.1 ± 7.3	36.9 ± 17.7
			NS	2	28 ± 3 (26–30)	86.5 ± 5.9	−0.08 ± 0.12	56.9 ± 3.0	65.3 ± 16.4	58.9 ± 8.0	55.0 ± 24.4
D2	14	230	LONG	27	169 ± 50 (55–254)	104.0 ± 10.2	−0.40 ± 0.19	25.0 ± 4.9	50.7 ± 11.6	21.7 ± 3.0	35.8 ± 8.2
			SHORT	26	34 ± 3 (29–42)	104.8 ± 9.0	−0.31 ± 0.18	26.8 ± 2.9	61.5 ± 15.0	26.7 ± 7.4	40.2 ± 14.9
			NS	19	44 ± 15 (17–73)	105.2 ± 12.3	−0.15 ± 0.20	74.4 ± 12.2	76.5 ± 9.8	48.6 ± 12.6	57.1 ± 25.2
D3	14	288	LONG	25	167 ± 41 (30–220)	106.2 ± 4.6	−0.51 ± 0.71	29.3 ± 6.5	67.1 ± 14.8	26.5 ± 5.2	46.2 ± 14.7
			SHORT	27	38 ± 10 (26–69)	108.5 ± 6.3	−0.25 ± 0.16	36.4 ± 11.9	72.9 ± 15.1	33.0 ± 12.8	50.0 ± 20.4
			NS	27	45 ± 15 (23–82)	107.0 ± 6.9	−0.13 ± 0.15	94.7 ± 14.4	95.2 ± 15.0	54.6 ± 18.4	76.3 ± 23.7

All trials were performed using operant conditioning, and participation by each dolphin was voluntary. Thus, each individual animal was not restrained and could refuse to participate or withdraw at any point during the experiment as previously detailed (Fahlman et al., 2019a, b; Fahlman et al., 2020b). Approximately 6 months before trials began (October 2017), the dolphins were desensitized and conditioned for the procedure. Trials were conducted on 6 separate occasions; April and September 2018, January, April and November 2019, and January 2020. Two dolphins (D2 and D3, Table 1) participated during all trial dates and one in only 2 out of the 6.

Breath-holds were separated into one of 3 categories; 1) LONG (120–180 s), 2) SHORT (30 s) or 3) no symbol (NS), where the dolphin decided the apnea duration. The LONG or SHORT breath-holds were differentiated by showing the dolphin a different symbol (cross or square) 5–10 s before the breath-hold. The duration of the LONG breath-hold was determined to maximize duration within each animal’s comfort. This assured that most LONG breath-holds were completed to the pre-determined duration and those that ended early were discarded. The duration of the SHORT breath-hold was long enough to be similar to a common inter-breath interval (Fahlman et al., 2015, 2017, 2020a; Cauture et al., 2019). The start and end of a breath-hold was the time from the last breath until the first breath following the breath-hold. While the duration from the start of the breath-hold until the stimulus to end the breath-hold was pre-determined, the duration of LONG or SHORT breath-holds were not always the same as the duration until the dolphin took its first breath varied.

Each trial consisted of an animal floating stationary in the water, dorsal side up and rostrum on deck with blow-hole out of the water. The trainer gave the dolphin a discriminative stimulus (SD) to turn its ventral side up to allow placement of 3 ECG electrodes on the sternum (Bickett et al., 2019; Cauture et al., 2019; Fahlman et al., 2019b, 2020b). When the ECG signal was confirmed, the animal was given an SD to turn to floating dorsal side up, and the ECG signal was again verified. The dolphin recovered for 3–5 minutes before the next breath-hold. During this time, the pre-dive ECG was recorded while the dolphin was inactive next to the trainer. Next, the trainer showed the dolphin the symbol for a LONG or SHORT breath-hold (a cross or a square, respectively), and presented the breath-hold SD to the dolphin to begin the dive. The breath-hold was terminated when the trainer presented an acoustic SD. For the NS breath-holds, the dolphin was only given the SD to begin the breath-hold, and the end of the dive was determined by the dolphin. Thus, the breath-hold duration for NS dives was variable (Table 1). All breath-holds analyzed in the current study were with the dolphin in a ventral up position. For each trial, the dolphin participated in up to 6 repeated dives (range: 1–6), with at least 3 minutes between each breath-hold. Each repeated dive was randomly selected from the 3 different types of breath-holds (no breath-hold type was repeated more than 3 times in a row). The randomization was set by a predetermined schedule. The number of repeated dives was dependent on the cooperation and behavior of the dolphin. Thus, not all breath-hold types were performed during each trial.

The ECG was recorded at 400 Hz using a data acquisition system (Powerlab 8/35, ADInstruments, Colorado Springs, CO, United States), and displayed on a computer running LabChart (v. 8.1.13, ADInstruments, Colorado Springs, CO, United States). The ECG was analyzed using the heart rate detection software in LabChart which automatically detected the R-peaks, using the following settings; typical QRS width = 80 ms, R-waves = 300 ms, pre-P baseline = 120 ms, maximum PR = 240 ms, maximum RT = 400 ms. Following the automatic detection, the R-peaks were manually verified and the i*f*_H_ determined from the time between R-R peaks. Noisy sections, or possible beats that did not contain a clear R-peak were removed.

The study protocols were evaluated and approved by the Animal Care and Welfare Committee at the Oceanogràfic (OCE-17-16, amendments OCE-29-18 and OCE-3-19i) and the Bureau of Medicine (BUMED, NRD-1015).

### Analysis

The focus of the current study was to evaluate if the *f*_H_-response could be conditioned by anticipation. We did this by showing a symbol for the expected dive duration (LONG or SHORT) with the hypothesis that anticipation would condition the *f*_H_-response.

The pre-dive *f*_H_ was the average *f*_H_ 60 s before the breath-hold (Pre). As past studies in the bottlenose dolphin have shown that *f*_H_, stroke volume, and cardiac output decreases with longer dive duration (Fahlman et al., 2019b), the minimum instantaneous *f*_H_ (i*f*_Hmin_) during the breath-hold, and the average instantaneous *f*_H_ (i*f*_Hend_) over the last 10 s of the breath-hold, were evaluated and expected to be lower for longer dives. To assess the anticipatory, or conditioned response, we compared the rate of change in instantaneous *f*_H_ (di*f*_H_/dt), the average instantaneous *f*_H_ (i*f*_Hstart_), and the minimum instantaneous *f*_H_ (i*f*_Hstartmin_) during the first 20 s of the breath-hold. We found considerable variation in the *f*_H_-response between trial types. For example, instantaneous *f*_H_ (i*f*_H_) changed from 90 to 100 beats ⋅ min^–1^ to around 35–40 beats ⋅ min^–1^ within 3–4 beats in some cases while in others it never went lower than 60 beats ⋅ min^–1^ even after 60 s. To account for this non-linear decrease in *f*_H_, i.e., di*f*_H_/dt, both the time and i*f*_H_ were log_10_-transformed to provide a linear fit to all i*f*_H_ over the first 20 s of the breath-hold. We then fitted a line to the data for log_10_-transformed *f*_H_ and time and used the slope as an index of the rate of change in *f*_H_, i.e., log_10_(di*f*_H_/dt).

### Statistical Analysis

We compared the *f*_H_ data within and between individuals. The relationship between a dependent variable (i*f*_Hmin_, di*f*_H_/dt, i*f*_Hstart_, i*f*_Hstartmin_ and i*f*_Hend_), and the experimental covariates, *M*_b_, type of breath-hold (LONG, SHORT, NS), months of conditioning, and the dive number in a session (DiveNo) were analyzed using linear-mixed effects models (function *lme*, R: A Language and Environment for Statistical Computing, R Foundation for Statistical Computing, version 3.6.1, 2019). While age may play a role in heart function, the current data did not suggest such a pattern. However, the number of animals we used was too small to assess this appropriately, so we do not consider this variable in our comparisons here. When the effect for type of breath-hold warranted inclusion, i.e., improvement in fit, we added the factor (LONG, SHORT, or NS) in the equation indicating the actual type by setting it to 1 while setting the others to 0. The individual animal was treated as a random effect, which accounted for the correlation between repeated measurements on the same individual (Littell et al., 1998). Initially, a univariate analysis on each independent variable was performed; only those variables with a *P*-value < 0.20 (Wald’s tests) were then considered in a multivariate analysis. Cross terms were considered to determine possible interaction effects in the relationship between the dependent variable and the covariates. The parameters from the most parsimonious model were chosen by the log-likelihood (LL) ratio test. Normality for all models was confirmed by the Bartlett test, and in case of unequal variances the variable was log_10_-transformed and normality confirmed. Acceptance of significance was set to the *P* < 0.05 level. Data are presented as the mean ± standard deviation, unless otherwise stated.

## Results

### Resting *f*_H_

The pre-apnea resting *f*_H_ [pre-*f*_H_, average *f*_H_ (± s.d.) 99.6 ± 11.5 beats ⋅ min^–1^, range: 86–108 beats ⋅ min^–1^] did not vary systematically with the type of dive (χ^2^ = 2.03, df = 2, *P* > 0.3), the number of repeated dives in a session (χ^2^ = 1.94, df = 1, *P* = 0.16), the number of dives during a day (χ^2^ = 1.72, df = 1, *P* = 0.19), or the number of months of conditioning (χ^2^ = 0.73, df = 1, *P* = 0.39). The base 10 logarithm transformed pre-*f*_H_ [log_10_(pre-*f*_H_)], was correlated with log_10_-transformed *M*_b_ (χ^2^ = 5.43, df = 1, *P* = 0.02, Table 2).

**TABLE 2:** Statistical results for different models assessing the variation in heart rate (*f*_H_).

Dependent	b_0_	log_10_(*M*_b_)	Dive type		DiveNo	*P*-value
			SHORT	NS		
log_10_(pre-*f*_H_)	3.14 ± 0.46	0.48 ± 0.19	–	–	–	=0.02
log_10_(di*f*_H_/dt)	−0.343 ± 0.059	–	0.171 ± 0.063	0.384 ± 0.070	−0.057 ± 0.017	<0.01
i*f*_Hstart_	55.3 ± 4.5	–	6.6 ± 2.7	24.3 ± 2.9	1.7 ± 0.7	<0.01
log_10_(i*f*_Hstartmin_)	1.46 ± 0.03	–	0.056 ± 0.018	0.47 ± 0.02	–	<0.01
log_10_(i*f*_Hmin_)	1.38 ± ± 0.02	–	0.08 ± 0.02	0.31 ± 0.02	–	<0.01
log_10_(i*f*_Hend_)	1.63 ± 0.04	–	0.04 ± 0.02	0.24 ± 0.03	−0.013 ± 0.006	=0.01

*The models include pre-apnea heart rate (pre-*f*_*H*_), the rate of change in instantaneous *f*_*H*_ (di*f*_*H*_/dt), the minimum instantaneous *f*_*H*_ (i*f*_*Hstartmin*_), and the average instantaneous *f*_*H*_ (i*f*_*Hstart*_) during the first 20 s of the breath-hold, and the minimum instantaneous *f*_*H*_ during the whole dive (i*f*_*Hmin*_) or the average instantaneous *f*_*H*_ during the last 10 s of the breath-hold (i*f*_*Hend*_). Some variables were transformed using the base 10 logarithm (log_10_) (see text for details). In these models, LONG is included in the default model and the factors SHORT (or NS) is 1 for a dolphin doing a SHORT breath-hold and 0 otherwise, and DiveNo is the number of randomly assigned breath-holds in a given session before the given dive. For example, if a SHORT dive was the second dive in a session, the predicted log_10_(di*f*_*H*_/dt) would be: [log_10_(di*f*_*H*_/dt)] = −0.343 + 0.384 × 0 + 0.171 × 1 − 0.057 × 2 = 10^–0.286^ beats ⋅ min^–1^ ⋅ s^–1^.*

### Heart Rate Responses During Apnea

**FIGURE 1:**
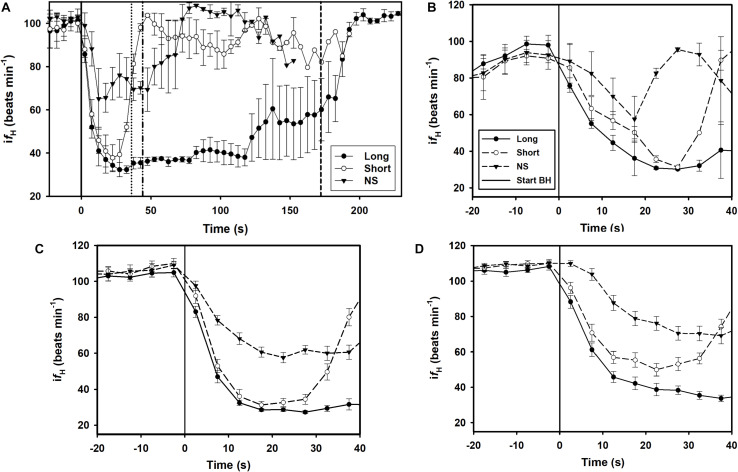
Instantaneous heart rate (i*f*_H_) against time before (time –20 to 0) and up to A) 230 s or 40 s during a breath-hold for dolphin B) D1, C) D2, D) D3 (Table 1). Data are average (±s.e.m., *n* = 3) i*f*_H_ for LONG (*n* = 58) and SHORT (*n* = 58) dives that were preceded by a symbol and with pre-determined dive duration, and dives without a symbol (NS, *n* = 48) in which dive duration was determined by the dolphin. **(A–D)** The solid vertical black line is the start of the breath-hold (BH) which is when the dolphin took the last breath before submerging. **(A)** The average time for the end of the breath-hold, the time of the first breath after the breath-hold, is indicated as broken vertical lines for the SHORT, NS and LONG dives (from left to right).

**FIGURE 2:**
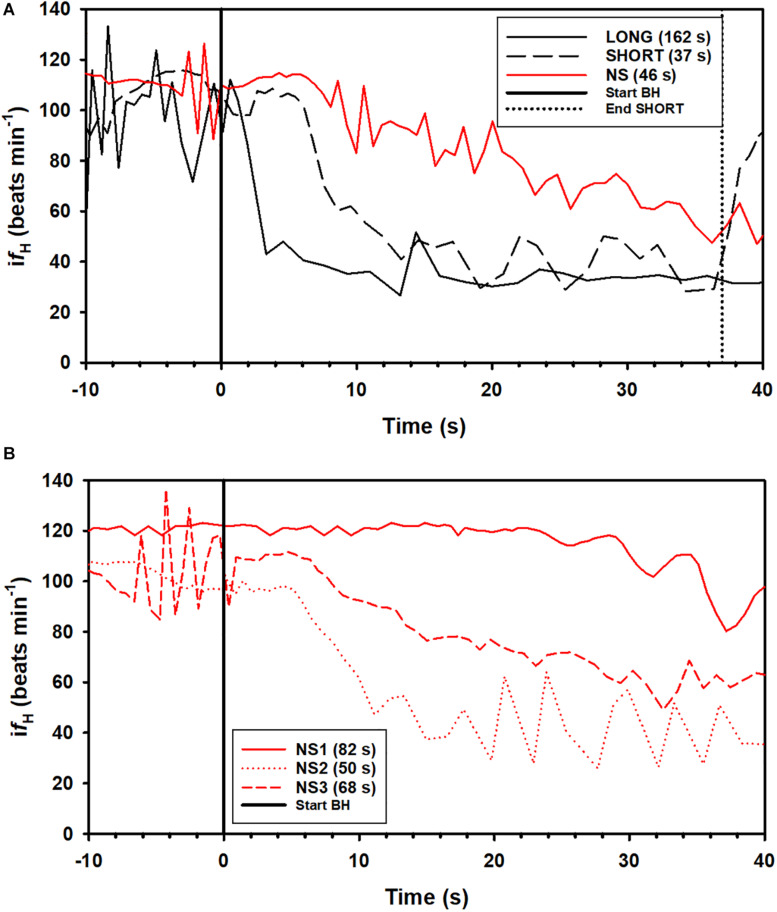
Examples of instantaneous heart rate (i*f*_H_) responses from individual dolphins against time before (time –20 to 0) and up to 40 s (time 0 to 40 s) during a breath-hold. **(A)** i*f*_H_ for a LONG and SHORT dive (each preceded by a symbol with a pre-determined dive duration), and a dive without a symbol (NS), where dive duration was determined by the dolphin. **(B)** i*f*_H_ variation during a breath-hold for 3 NS dives in dolphin D3 (Table 1). The dive durations, the time from the last breath before and first breath after a breath-hold is indicated in parenthesis.

A total of 164 breath-holds were analyzed, ranging from 26 s to 254 s (Table 1). The *f*_H_-response for all trials was binned every 5 s from 10 s before the breath-hold began to the end of the trial (Figure 1A) or the first 40 s of the dive for each individual dolphin (Figures 1B–D). Figure 2A shows examples of *f*_H_ for the 3 different types of breath-hold, and Figure 2B the *f*_H_-responses during NS breath-holds of different durations.

The di*f*_H_/dt (Bartlett *K*^2^ = 117, *P* < 0.01, df = 2), i*f*_Hmin_ (Bartlett *K*^2^ = 69, *P* < 0.01, df = 2), i*f*_Hend_ (Bartlett *K*^2^ = 6.44, *P* = 0.04, df = 2), and i*f*_Hstartmin_ (Bartlett *K*^2^ = 22.4, *P* < 0.01, df = 2), but not i*f*_Hstart_ (Bartlett *K*^2^ = 0.2, *P* > 0.9, df = 2), all had unequal variances between groups (LONG, SHORT, NS) and were therefore log_10_-transformed.

To assess whether the rate of change in i*f*_H_ during the first 20 s of the apnea was anticipatory, we compared the log_10_(di*f*_H_/dt) between the different dives; short (SHORT) and long (LONG) when breath-hold duration was pre-determined and apneas were initiated with a symbol, or when the dolphins decided the duration following only an SD to begin the apnea (NS). Neither *M*_b_, nor number of months since the apnea conditioning had begun (*P* > 0.1 for all), warranted inclusion in any model. The log_10_(di*f*_H_/dt) increased with repeated trials within a session, and differed significantly between groups (χ^2^ = 11.0, df = 1, Table 2). The Tukey’s *post hoc* test showed that there were significant differences for log_10_(di*f*_H_/dt) for all groups: LONG-SHORT (*Z*-value = 2.75, *P* = 0.016), LONG-NS (*Z*-value = 5.55, *P* < 0.001) SHORT-NS (*Z*-value = 3.19, *P* = 0.004).

The average *f*_H_ during the first 20 s of the breath-hold (i*f*_Hstart_) increased by an average of 3.3 beats ⋅ min^–1^ with each repeated trial during a session, i.e., DiveNo (χ^2^ = 15.1, df = 1, *P* < 0.01), and the most parsimonious model included both Dive type and DiveNo (χ^2^ = 70.5, df = 2, *P* < 0.01, Table 2). A Tukey’s *post hoc* test showed that there were significant differences for i*f*_Hstart_ for all group comparisons: LONG-SHORT (*Z*-value = 2.53, P = 0.03), LONG-NS (*Z*-value = 8.45, *P* < 0.001) SHORT-NS (*Z*-value = 6.47, *P* < 0.01). The minimum i*f*_H_ for the first 20 s (i*f*_Hstartmin_) was significantly different between dive types (χ^2^ = 281, df = 2, *P* < 0.01, Table 2), with significant differences between LONG-NS (*Z*-value = 26.0, *P* < 0.01), and SHORT-NS (*Z*-value = 22.6, *P* < 0.01) dive types, but not between LONG-SHORT (*Z*-value = 3.06, *P* > 0.05, Table 1).

Both log_10_(i*f*_Hmin_) (χ^2^ = 114.0, df = 2, *P* < 0.01) and average log_10_(i*f*_Hend_) also differed between dive types, and average log_10_(i*f*_Hend_) also decreased with repeated trials (DiveNo) within a session (χ^2^ = 5.50, df = 1, *P* = 0.01, Table 2). Tukey’s *post hoc* tests showed that for log_10_(i*f*_Hmin_) there were significant differences between all dive types [LONG-NS (*Z*-value = 13.3 *P* < 0.01), LONG-SHORT (*Z*-value = 3.80, *P* < 0.01), and SHORT-NS (*Z*-value = 10.1, *P* < 0.01, Table 2)], but for log_10_(i*f*_Hend_) there were no differences between SHORT and LONG dive types [LONG-NS (*Z*-value = 9.1, *P* < 0.01), LONG-SHORT (*Z*-value = 1.8, *P* > 0.1), and SHORT-NS (*Z*-value = 7.7, *P* < 0.01, Tables 1, 2].

## Discussion

In our study, we showed that anticipation of a LONG breath-hold caused a faster reduction in *f*_H_ (di*f*_H_/dt) to a lower average (i*f*_Hstart_), and minimum *f*_H_ (i*f*_Hstartmin_) during the first 20 s of the breath-hold than anticipation of a SHORT breath-hold. In addition, the average minimum *f*_H_ (i*f*_Hmin_) or average *f*_H_ during the last 10 s of the breath-hold (i*f*_Hend_) were considerably lower during both LONG and SHORT breath-holds as compared with NS breath-holds (Table 2). As it is known that *f*_H_ tends to decrease temporally during the apnea (Fahlman et al., 2019b), the difference between LONG and NS was not unexpected. However, the i*f*_Hmin_ and i*f*_Hend_ were 70% and 50% higher, respectively, during NS as compared with SHORT breath-holds, even though the former was on average 22% longer in duration. We propose that these results indicate a conditioned response that allows dolphins to regulate *f*_H_ during diving, likely to allow *selective gas exchange* (Hodanbosi et al., 2016; García-Párraga et al., 2018).

### Evidence for Conditioned Control of Heart Rate

While it is clear that the dive response is exhibited by all vertebrates studied and that the response is heritable, its actual role and regulation within and between species is debated (Mottishaw et al., 1999; Fahlman et al., 2011; Ponganis, 2011; Ponganis et al., 2017; Parkes, 2019). A number of factors such a facial immersion (in humans), breathing frequency, tidal volume, age, blood O_2_ and CO_2_ tension, blood pressure, and emotional state may explain the large variability within and between individuals (Irving, 1963; Harrison et al., 1972; Lin et al., 1972; Jones et al., 1973; Moore et al., 1973; Angell-James et al., 1978, 1981; Openshaw and Woodroof, 1978; Blix, 1987; Bernardi et al., 1989; Thompson and Fedak, 1993; Castellini et al., 1994a, b; Andrews et al., 2000; Fahlman et al., 2011; Elmegaard et al., 2016, 2019; Kaczmarek et al., 2018; Cauture et al., 2019). In addition, previous studies have suggested that at least some marine mammals can be conditioned to alter the *f*_H_ response depending on anticipation (Elmegaard et al., 2016; Kaczmarek et al., 2018), possibly due to suprabulbar or cortical influences (Butler and Jones, 1982, 1997; Panneton, 2013). If the *f*_H_-response can be conditioned and varies due to anticipation, it may help explain some of the large variability between forced diving, where the response is maximal and heritable, versus those during voluntary or freely diving mammals (Blix, 1987; Mottishaw et al., 1999; Fahlman et al., 2011). To further investigate the magnitude of this conditioned control, we investigated variation in the *f*_H_-response in the bottlenose dolphin during 3 different types of diving. While assessment of heart rate variability (HRV) may be considered an interesting method to separate the autonomic components of the cardiovascular plasticity observed in the current study, such analysis could not be conducted due to the dynamic conditions of these experiments (see Supplementary Material).

Trials in the current study consisted of repeated breath-holds, a minimum of 3, with brief intermittent apneas to attach and remove the ECG electrodes, and 1–4 additional breath-holds with sufficient surface time to assure that the dolphin had fully recovered based on past studies (Fahlman et al., 2019a, b). This allowed us to focus the experiment on nervous control of heart function. Similar conditioned cardiovascular responses have been seen in other species (Kooyman and Campbell, 1972; Elmegaard et al., 2016; Kaczmarek et al., 2018), which supports the hypothesis that the changes in *f*_H_ and blood flow are part of the repertoire of adaptations that conserve available O_2_, maximize efficiency and extend aerobic dive duration (Davis and Kanatous, 1999).

The breath-hold trials in the current study were performed under voluntary control, and the dolphin could end a breath-hold at their own volition. During forced dives, the change in *f*_H_ is generally rapid, possibly because the animal prepares more conservatively for a dive duration of unknown duration (Scholander, 1940; Kooyman and Campbell, 1972; Kooyman, 1985; Fahlman et al., 2011). The change in *f*_H_ during conditioned dives can also be rapid (Elsner et al., 1966; Ridgway et al., 1975; Houser et al., 2010), but was much more variable when the animal was able to determine the dive duration (Figure 1). Thus, the rapid *f*_H_-response observed in the current study was unlikely a maximal bradycardia as seen during a forced breath-hold (Elsner, 1965; Butler and Jones, 1982), and the dive duration was much shorter than the maximal duration seen in this species (Fahlman et al., 2018). Thus, the symbols for a LONG and SHORT breath-hold allowed the dolphin to anticipate the duration and adjust the physiological response. Interestingly, similar *f*_H_-responses, in which the reduction in *f*_H_ was delayed, were also reported in the harbor porpoise, harbor seal and California sea lion depending on the condition (Elmegaard et al., 2016; Kaczmarek et al., 2018).

When shown a symbol for a dive, the di*f*_H_/dt, i*f*_Hstart_, i*f*_Hmin_, and i*f*_Hend_ differed between groups in a way that appeared to maximize the aerobic dive duration. Furthermore, the results for the di*f*_H_/dt, and i*f*_Hstart_ suggested that the dolphins prepared for an anticipated apnea by reducing the *f*_H_ to a lower average value more rapidly, and to a lower level within the first 20 s of a LONG as compared with both the SHORT and NS breath-holds. Even though the average breath-hold duration was longer during NS as compared with the SHORT breath-hold, the rate of change in *f*_H_ (di*f*_H_/dt) during the first 20 s for NS dives was less pronounced, and the minimum *f*_H_ (i*f*_Hstart_) was higher when dolphins determined the duration of their breath-holds themselves. In addition, when the dolphin determined the breath-hold duration, the *f*_H_ was more variable within and between individuals and trials, suggesting an ability to adjust the *f*_H_ response depending on the expected situation.

In the current study we used a randomized design, where symbols and SDs were used to communicate expectations, which clearly showed that the conditioned *f*_H_-response depends on the anticipated task. The dolphin was not shown what type of dive was expected until 5–10 s before the breath-hold. In addition, the pre-apnea and diving *f*_H_‘s were within the range of those reported in previous studies in conditioned bottlenose dolphins (Noren et al., 2004; Fahlman et al., 2019b, 2020b), and did not vary depending on the type of breath-hold (Figure 1). While the *f*_H_-response during the breath-hold in the current study is similar to that reported in the harbor porpoise, the pre-dive *f*_H_ varied considerably in the harbor porpoise (Elmegaard et al., 2016, 2019). It is possible that a non-random design, where the animal anticipated the task beforehand resulted in changes in the breathing frequency or tidal volume, which are known to alter *f*_H_ (Cauture et al., 2019; Fahlman et al., 2019b, 2020b). In the current study, the symbol was shown immediately before the breath-hold which may explain why there were no differences in pre-diving *f*_H_.

In most LONG or SHORT breath-holds, there was a rapid reduction in *f*_H_ immediately as the dolphin submerged (Figures 1, 2A). For the NS breath-holds, on the other hand, the di*f*_H_/dt was substantially lower (Figures 1, 2) with most of these dives showing a very slow decline in *f*_H_, even for NS breath-holds up to 80 s (Figure 2B). In addition, there was considerably more variation in the *f*_H_ during the first 20 s into the breath-hold in the NS group (Figures 1, 2B), as compared with either LONG or SHORT breath-holds. The *f*_H_ for some NS breath-holds only began declining after 10–30 s into the breath-hold (Figure 2B, NS1), while for others it was faster but did not approach the diving *f*_H_ of 35–45 beats ⋅ min^–1^, that is common in this species, and was reached during most LONG or SHORT breath-holds (Figures 1, 2B, NS2). Yet in other NS breath-holds, there was rapid variation in i*f*_H_, with arrhythmias changing between two set points (Figure 2B, NS3), similar to that seen in the gray seal (see Figure 6A in Thompson and Fedak, 1993). We have commonly seen this variation and arrhythmias in cetaceans such as the bottlenose dolphin, short-finned pilot whale (*Globicephala macrorhynchus*), killer whale (*Orcinus orca*), false killer whale (*Pseudorca crassidens*), and beluga (*Delphinapterus leucas*), and agree with the suggestion that these arrhythmias are common in marine mammals and do not represent cardiovascular morbidity (Ponganis et al., 2017; Arya et al., 2018; Bickett et al., 2019; Fahlman et al., 2019b, 2020b). Rather, these changes in *f*_H_ and arrhythmias provide further evidence of extensive cardiovascular plasticity in cetaceans.

While there were no differences in the diving *f*_H_ with repeated dives, the i*f*_Hstart_ increased by 3%, while the di*f*_H_/dt, and i*f*_Hend_ decreased by 13 and 4%, respectively, with each repeated breath-hold in a trial. Thus, the dolphin had a faster reduction in *f*_H_, but to a slightly higher minimum *f*_H_ during the first 20 s, during repeated dives.

### Neural Basis for Central Regulation of the Heartbeat

The results of our study showed that dolphins are able to regulate (slow down) *f*_H_ immediately after (within 5 s, Figure 1) the external stimulus (breath-holding and then immersion) induces the dive response. The immediate reduction in *f*_H_ (Figure 1) was more marked in the two experimental groups (LONG and SHORT), but also present in the control group (NS). This immediate reduction in *f*_H_ in response to the stimulus could have been caused by one of several possibilities: (a) voluntary control, i.e., direct somatic motor control; (b) induction by internal clues; (c) induction by external clues. A direct somatic control of the frequency of heart contraction, or *voluntary control*, implies that the pyramidal (in humans) or extrapyramidal (in dolphins) motor systems were able to regulate cardiac muscle activity through a (late cervical or early thoracic) spinal nerve, a scenario that goes against the current understanding of the organization of the central nervous system in healthy mammals (see Figure 6.52 in Cozzi et al., 2017), and is therefore unlikely. Furthermore, somatic control of the *f*_H_ would also require the presence of motor plaques in the heart, a mixture of cardiac and striated muscle fibers that have not been found in any mammal. To avoid confusion or overinterpretation of the results, we use the word *conditioned* instead of the more ambiguous *cognitive*, a term that may induce the reader to believe that the brain has the direct capacity to modify *f*_H_ by acting on (non-existing) motor plaques. The control of breathing and blood pressure remains a midbrain prerogative (for thorough review see Ghali, 2020), and its variations depend on complex - and fast - induced reflex circuits.

Changes to *f*_H_ may also be induced by internal factors, including pH of the blood O_2_ and CO_2_ levels, but these are generally much slower to respond than what we observed for the LONG and SHORT *f*_H_-responses (Kooyman and Campbell, 1972). The slower reduction in *f*_H_ during NS dives could reflect autonomic adjustment of peripheral and central chemoreceptors as the blood O_2_ tension decreases and the CO_2_ tension increases (Figures 1, 2; De Burgh Daly et al., 1977; Elsner et al., 1977). Finally, a variation of *f*_H_ may be induced by external cues. A potential explanation is that the conditioned dolphins recognize the visual symbol, which is mediated by the visual cortex and act through relays involving the cingulate cortex and the amygdala and finally reaching the medial forebrain bundle (for general reference see Kandel et al., 2013), with a vagal effect on cardiac centers in the brainstem, one of the main pathways involved in the central regulation of visceral functions. This more plausible and alternative mechanism for control of *f*_H_ would shift the focus from the somatic motor system to the limbic system and its influence on visceral functions.

Specific circumstances, including listening to music (Yuen and Sander, 2017) and meditation (Arya et al., 2018; Ionson et al., 2019) may affect *f*_H_ by this route in humans, but such vagal relaxation are relatively slow to set in. Even under these circumstances the control of *f*_H_, although decided by the individual, is not induced by direct somatic motor control, i.e., is not *voluntary*. Often such reactions take a while to set in, but visceral functions can be affected much quicker if necessary. For example, a similar pathway is used in the immediate *f*_H_ increase in a threatening situation. Cetaceans clearly have evolved to rapidly decrease *f*_H_ in a similar way, which is a more suitable reaction to a threat in a diving mammal that needs to preserve oxygen for long dives when escaping predators. Our study, and a previous one on porpoises (Elmegaard et al., 2016), show that these decreases can be modified in their strength by learning, even in stress-free situations. The most parsimonious learning mechanism involved here is instrumental conditioning, which does not require cognition (Bayne et al., 2019), but is different from the control over the onset of a reflex as in classical conditioning. It is questionable whether cognitive control beyond association as suggested by Elmegaard et al. (2016) is required for these adjustments. In the wild, changes in *f*_H_ may be induced by acoustic signals or echoes revealing the presence of prey in the water column.

## Conclusion

In our study, we showed a conditioned response in the bottlenose dolphin, in which the cardiac response depended on the type or length of the dive (Figure 1). While the dolphins participated voluntarily in all trials, the symbol for a LONG and SHORT breath-hold indicated an expected breath-hold duration. With the longer breath-hold duration, the rate of change in *f*_H_ (di*f*_H_/dt) was greater, and the average *f*_H_ during the first 20 s of the breath-hold (i*f*_Hstart_) was lower for LONG as compared with SHORT breath-holds, suggesting that the dolphin anticipated a longer hypoxic period. Without a symbol that indicated a certain breath-hold period, the *f*_H_-response was more varied, and for most of these apneas there was a slow decline from the pre-dive *f*_H_ throughout the breath-hold period. We propose that the cardiorespiratory changes in the dolphin suggest a capacity to slow *f*_H_ induced by external cues and subsequent activation of the limbic system to affect vagal cardiac centers in the brainstem. While an ability to regulate *f*_H_ is known in humans, such as meditating yogis (Elsner, 2015), where a reduction in *f*_H_ may take 30 minutes as they enter their trance, the dolphin is doing the same within seconds and to a much greater extent.

## Data Availability Statement

The datasets presented in this study can be found in online repositories. The names of the repository/repositories and accession number(s) can be found below: The data used in this study are available from OSF: https://osf.io/t8jry or upon request to AF, afahlman@whoi.edu.

## Ethics Statement

The animal study was reviewed and approved by Animal Care and Welfare Committee at the Oceanogràfic (OCE-17-16, amendments OCE-29-18 and OCE-3-19i), and the Bureau of Medicine (BUMED, NRD-1015).

## Author Contributions

AF, MMan, SJ, VJ, MMal, BC, and AB: conceptualization. AF, MMan, SJ, AB: methodology. AF and MMal: formal analysis. AF, MMan, SJ, AB: investigation. AF: validation, resources, data curation, writing – original draft, supervision, project administration, and funding acquisition. MMan, SJ, VJ, MMal, BC, and AB: writing – review and editing. All authors contributed to the article and approved the submitted version.

## Conflict of Interest

AF was affiliated with the company Global Diving Research Inc., while completing this work but received no financial compensation. The remaining authors declare that the research was conducted in the absence of any commercial or financial relationships that could be construed as a potential conflict of interest.
