# Supplementary material for: Conditioned Variation in Heart Rate During Static Breath-Holds in the Bottlenose Dolphin (Tursiops truncatus)
Source: Front Physiol. 2020 Nov 24;11:604018. doi: 10.3389/fphys.2020.604018 (PMC7732665; doi:10.3389/fphys.2020.604018)
Supplement: Supplementary file 1 [file Data_Sheet_1.docx]

**Supplementary Information**

Possibly, it might be tempting to use the analysis of heart rate variability (HRV) to elucidate the details of heart period changes during these experiments (Task Force of the European Society of Cardiology the North American Society of Pacing Electrophysiology, 1996). There are, however, several problems with such an approach.

Firstly, with the exception of the so-called normalised (or quasi-normalised) spectral components of HRV, the HRV indices are all heart rate dependent (Malik et al., 2019). Because of the different heart rate responses to the individual phases of the study (see the Supplementary Figure below) we would find it difficult if not impossible to separate the heart rate influence from the heart-rate independent regulation mechanisms.

Secondly, normalised spectral components require stable conditions without any heart rate trends and, at least in human and canine physiology, also require such stable conditions to be maintained for a period of some minutes so that the spectral analysis leads to interpretable results (Task Force of the European Society of Cardiology the North American Society of Pacing Electrophysiology, 1996). This was clearly not achievable during the experiments described here as also shown in the Supplementary Figure.

Finally, while models of heart rate regulation based on HRV have been developed and tested in human studies (and confirmed also in other mammal species), these models explain the changes in cardiac periods based on autonomic feed-back mechanisms (Task Force of the European Society of Cardiology the North American Society of Pacing Electrophysiology, 1996; Malik et al., 2019). In more detail, the feed-back mechanism models are based on the fact that vagal reflexes are faster compared to the sympathetic reflexes. Indeed, in man or in dog, vagal activity or withdrawal can influence the immediately subsequent cardiac cycle but responses to sympathetic activity occur with an initial delay of around 5 seconds and reach the maximal effect after approximately 20 to 30 seconds. If the autonomic tone is neither completely abolished nor fully saturated, these different response speeds lead to different modulation frequencies. This allows employing engineering methods of spectral analysis to estimate the extent of vagal and sympathetic modulations. Under normal circumstances, the extent of such modulations expresses, albeit only approximately, the vagal and sympathetic tone. Moreover, vagal modulations are mostly synchronised with the respiration-driven intrathoracic pressure changes.

It is not obvious whether these HRV principles are directly applicable also to aquatic mammals with substantially different respiration periodicity (Fahlman et al., 2017). Also, in human physiology, cortex-based regulations are known to be possible through vagal withdrawal (e.g. due to fear or other abrupt mental shocks). However, fast acting cortex-based vagal boosts, resulting in the rapid heart rate deceleration observed in the current study, are little known in human physiology. This suggests that for dolphins, and perhaps also other diving animals that frequently experience apnea episodes, different mechanisms, and thus different models of heart period regulation might need to be considered. For this reason, detailed studies of cardiac periodicity in aquatic mammals seem to be warranted. Among other possibilities, measurements of acceleration and deceleration capacity (Bauer et al., 2006) might be worth elucidating before concluding whether the standard HRV technology can be applied when investigating these animals.


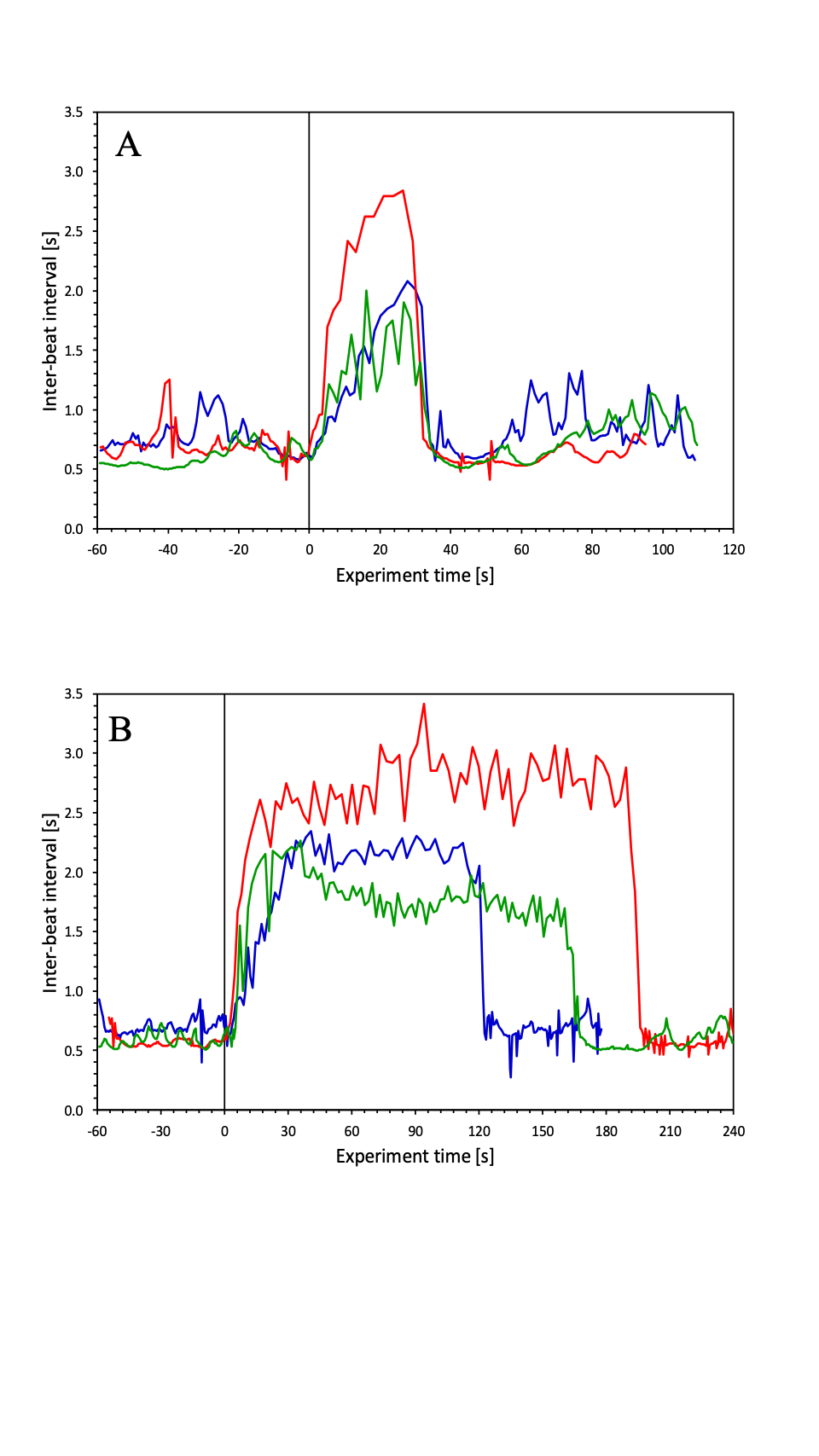


**Supplementary figure:** The panels show examples of the development of inter-beat intervals measured in separate individual experiments in three investigated animals (distinguished by different colours). The A) top and B) bottom panel corresponds to the “short” and the “long” experiments, respectively. The time zero correspond to the initial instruction given to the animals. The graphs are presented to show substantial data instability that precludes the use of spectral HRV methods.
